# On the Use of Alkalis and Absorbents in Disorders of the Stomach and Bowels

**Published:** 1802-10-01

**Authors:** 


### Medical and Physical Journal.

VOL. VIII.] OCTOBER 1, 1802. [NO. XLIV.

### *On the Use of Alkalis and Absorbents in Disorders of the Stomach and Bowels.*

THIS season of the year is generally productive of complaints in the first passages: But where the summer has been unusually hot, and fruit plenty, these disorders are observed to be more frequent, more severe, and more obstinate. Most of our readers, we conclude, have already observed the effects of the last summer, in the production of Cholera and Diarrhœa; and when the cold weather sets in, they will probably become more frequent.

As there are two methods of treating these complaints, almost diametrically opposite to each other, and as our experience has not been inconsiderable, we shall take this occasion of making a few observations on the subject, and subjoining others which have been made in America.

When a patient is suddenly attacked with vomiting or purging, or both, and these are attended with severe pains in the stomach and bowels, cold extremities, and general spasms; the *regular* mode of treatment is to get down warm water, chamomile tea, broths, weak brandy and water, &c. in considerable quantity, to dilute the acrid bile. As soon as the patient becomes tolerably easy, or obtains some respite from his misery, small doses of rhubarb are given in mint water, with nutmeg water, or some aromatic confection; and to these are often added paregoric elixir, laudanum, or chalk. This mode of treatment will generally succeed, and it appears as rational as it is regular; for, say its abettors, how can we cure a disease while the cause of it is within? This idea however of evacuating the offending bile, when it gets possession of young practitioners, very often leads them to the use of antimonial emetics and drastic purgatives, which we think highly improper and often dangerous.

Having often had occasion to see cases which had been left to the efforts of Nature, or treated with evacuants for several days, prove very obstinate, and sometimes degenerate into Dysenteries, we were induced to view this disease in a different light.

The general tendency to ſpaſm obſerved in it, the ill effects of acids, and, on the contrary, the good effects of abſorbents and aromatics, ſo conſtantly obſerved, even in primo morbi inſultu, induced us to try a plan of treatment the reverſe of the regular one. Inſtead of evacuating the bile, we gave ſmall doſes of opium and chalk with aromatics, every hour, till the patient became eaſy.

When the irritation and ſpaſmodic ſymptoms are ſubdued, which this plan never fails to effect in a few hours, if the extremities are kept warm, we have recourſe to proper clyſters to evacuate the bowels. If the patient continues free from any return of the attack for twelve or twenty hours, we give a moderate doſe of rhubarb and magneſia with fifteen grains of ſoda, and repeat it if neceſſary on the morrow.

In reading the Medical Tranſactions of America, we found that Dr. Mitchill's theory of ſepton or azotic acid being the cauſe of ſeveral epidemic diſeaſes, had led practitioners on that continent to treat Autumnal Diarrhœas on a ſimilar principle. Dr. Daniel, in a letter to Dr. Mitchill, dated March 29, 1802, ſays, "I have lately put to the teſt of practice your theory reſpecting morbid affections produced by ſeptic acid generated in the inteſtinal canal. As the reſult has been highly ſatisfactory, I take the liberty, though a ſtranger, of addreſſing you upon the ſubject.

"I think it neceſſary to premiſe, that, during the laſt ſummer and autumn, the inhabitants of this place, and of the country for ſome diſtance around, were nearly exempt from the uſual intermitting and remitting fevers of thoſe ſeaſons; and this exemption extended even to thoſe living near marſhes and water courſes. The only diſeaſe which engaged much of my attention was a diarrhœa of a ſingular kind, and that was ſo general that I believe there were few perſons who did not ſuffer more or leſs from it during the time of its prevalence.—I ſhall not trouble you with a minute deſcription of this complaint, but mention ſome of its moſt remarkable ſymptoms as ſufficient for my preſent purpoſe. The moſt uniform and characteriſtic feature was a very white, chalky appearance of the fæces; for in not one ſingle inſtance did I obſerve the ſlighteſt tinge of bile, but, on the contrary, a total abſence of that fluid. The dejections, though frequent, were not very liquid; and my patients complained, in paſſing them, of a hot, ſmarting ſenſation at the end of the rectum. The febrile ſymptoms were ſeldom high, and in ſome caſes entirely abſent, though the pulſe generally was ſtronger than would have been expected, conſidering the degree of languor and depreſſion of ſtrength which uſually attended. In ſome inſtances, where the appetite became very much impaired, and the ſtools very frequent, they were mixed with mucus tus and blood; but this was not a common occurrence. I think it remarkable that moſt of the affected perſons complained of uneaſy ſenſations in the region of the liver; but none of their ſkins diſcovered a bilious tinge.

"This diſeaſe was in very few inſtances fatal, but extremely obſtinate and diſtreſſing. It did not end with the autumn, like the complaints of that ſeaſon, but continued to ſhow itſelf till the end of January; ſince when I have not heard of it. I was myſelf amongſt the firſt attacked by it; and, in addition to the ſymptoms mentioned above, was troubled with frequent eructations of air, which diffuſed a very hot, keen, and diſagreeable ſenſation upon the fauces and mouth; but differing from what is uſually called heartburn.

"My firſt curative attempts upon this epidemic were very unſatisfactory indeed. I tried the whole liſt of remedies uſually given in ſimilar affections; ſuch as emetics, cathartics, anodynes, bitters, and aſtringents; but all to little purpoſe, the ſymptoms generally returning as ſoon as the action of the medicines was over.

"My chagrin at being ſo often baffled in my attempts to cure a diſeaſe attended with no very formidable ſymptoms, excited a cloſer inveſtigation of its nature; and happening to turn my attention to your letter to Dr. Percival, of Mancheſter, publiſhed in the firſt volume of the Medical Repoſitory, my perplexity reſpecting its exciting cauſe and treatment vaniſhed. Your reaſoning proved fully to my mind, that, from the nature of its uſual contents, there muſt be a conſtant diſpoſition to the evolution of ſeptic acid in the alimentary canal whenever the digeſtive powers are impaired; that nature has, in her bounty, given us a corrective of this proceſs in the alkaline quality of the bile; but whenever this corrective is wanting, the production of that acid muſt, of courſe, be the conſequence. This doctrine was certainly a happy one, when applied to the diſeaſe of which I have been ſpeaking. My earlieſt obſervations upon the ſymptoms convinced me that there was a torpor in the ſecreting powers of the liver, and a conſequent deficiency of bile; but this led me no further than to the uſe of bitters, &c. as ſubſtitutes for that fluid. It was reſerved for you, Sir, to point out the neceſſity of ſaturating the acid poiſon which aggravated the ſymptoms and protracted the cure. As ſoon as this neceſſity was impreſſed upon my mind, my difficulties were at an end; for, except giving calomel occaſionally, with a view of rouſing the liver, I had nothing more to do than to give, during the day, frequent doſes of carbonate of potaſh diſſolved in chamomile tea, and an anodyne at night. In delicate ſtomachs the alkali was joined with citric acid. Under this treatment the diſeaſe not only loſt its obſtinacy, but became a mere trifle; and I think my acknowledgments due to you for the relief which my feelings experienced on the occaſion.

"Before I cloſe this letter, I muſt beg leave to draw your attention to a fact which has come under my obſervation: It is the ſingular effect which opium had upon two gentlemen of my acquaintance. They are brothers, and their habits nearly the ſame. They have both informed me that opium, as is uſual, ſuſpends the action of their inteſtines for ſome time (never leſs than twenty-four hours), and then they are ſeized with a briſk diarrhœa, which continues from four to ſix hours. The fæces diſcharged at that time are quite white, or, to uſe their own words, "they reſembled thickened milk, except being rather more liquid." But the moſt remarkable circumſtance is, that the diſcharges are of ſo active a quality as to excite the moſt inſufferable pain, and completely excoriate the anus. One of them ſaid, "he did not think *aqua-fortis* would hurt him worſe," and the pain had often been ſo great as to bring out a clammy ſweat upon his ſkin and induce faintneſs.

"In attempting an explanation of this phenomenon, I have ſuppoſed the opium to poſſeſs, in thoſe two perſons, a ſpecific power of ſuſpending, for a time, not only digeſtion, but alſo the ſecretion and tranſmiſſion of bile to the ſtomach and inteſtines; that, during this ſuſpenſion, a conſiderable accumulation of ſeptic acid takes place, and excites a commotion in the inteſtines, which does not ceaſe until they are emptied; or, the liver ſhakes off its torpor, and throws in the neceſſary quantity of bile to correct the acid.

"To put this reaſoning, in ſome meaſure, to the teſt of experiment, I prevailed upon one of the gentlemen to ſubmit to the effects of a doſe of opium under my directions. Early in the evening, I gave him a doſe of a ſolution of ſal abſinthii, and at bed time I gave him another, with a grain of pure opium. He repeated the doſes of the ſolution ſeveral times during the fore part of the enſuing day, and in the afternoon it ſtimulated his bowels to gentle action. The ſtools were not quite ſo white as they had formerly been after taking opium, and entirely deſtitute of their ſharp, corroding quality.

"I think it would be ſuperfluous to offer any opinion upon this experiment, as the reſult appears to me to ſpeak plainly for itſelf.

"I believed the above facts to be corroborative of your theory, and have therefore troubled you with them, and I ſhould feel honoured in hearing that you concurred in that belief."

